# Modeling CaMKII in cardiac physiology: from molecule to tissue

**DOI:** 10.3389/fphar.2014.00009

**Published:** 2014-02-04

**Authors:** Birce Onal, Sathya D. Unudurthi, Thomas J. Hund

**Affiliations:** ^1^The Dorothy M. Davis Heart and Lung Research Institute, Wexner Medical Center, The Ohio State UniversityColumbus, OH, USA; ^2^Department of Biomedical Engineering, College of Engineering, The Ohio State UniversityColumbus, OH, USA; ^3^Department of Internal Medicine, Wexner Medical Center, The Ohio State UniversityColumbus, OH, USA

**Keywords:** calmodulin kinase II, mathematical modeling, calcium, arrhythmias, heart failure

## Abstract

Post-translational modification of membrane proteins (e.g., ion channels, receptors) by protein kinases is an essential mechanism for control of excitable cell function. Importantly, loss of temporal and/or spatial control of ion channel post-translational modification is common in congenital and acquired forms of cardiac disease and arrhythmia. The multifunctional Ca^2+^/calmodulin-dependent protein kinase II (CaMKII) regulates a number of diverse cellular functions in heart, including excitation-contraction coupling, gene transcription, and apoptosis. Dysregulation of CaMKII signaling has been implicated in human and animal models of disease. Understanding of CaMKII function has been advanced by mathematical modeling approaches well-suited to the study of complex biological systems. Early kinetic models of CaMKII function in the brain characterized this holoenzyme as a bistable molecular switch capable of storing information over a long period of time. Models of CaMKII activity have been incorporated into models of the cell and tissue (particularly in the heart) to predict the role of CaMKII in regulating organ function. Disease models that incorporate CaMKII overexpression clearly demonstrate a link between its excessive activity and arrhythmias associated with congenital and acquired heart disease. This review aims at discussing systems biology approaches that have been applied to analyze CaMKII signaling from the single molecule to intact cardiac tissue. In particular, efforts to use computational biology to provide new insight into cardiac disease mechanisms are emphasized.

## INTRODUCTION

Signal transduction, whereby a cell receives and processes extracellular information to coordinate a cellular process, is critical for normal cell function. Signal-transduction systems are commonly perturbed in disease, making core constituents (e.g., kinases) attractive therapeutic targets (Levitzki, 2003). While we have learned a great deal about the components of key signaling pathways, the complex nature of these vast networks represents a significant obstacle to understand their dynamics, regulation, and function. Systems biology and computational modeling of biological systems have become increasingly valuable in enhancing our understanding of these complex protein interaction networks.

Systems biology involves the study of the complex interactions and associated dynamics found in biological systems. Systems biology approaches commonly involve translation of the system into a mathematical model for subsequent computer simulation and analysis. As systems-based approaches have gained favor in the study of human disease processes, so has mathematical modeling of biological systems with associated advancements in understanding complex biological phenomenon like circadian rhythms, apoptosis, synaptic plasticity, and cell communication (Herzel and Bluthgen, 2008; Kotaleski and Blackwell, 2010).

**FIGURE 1:**
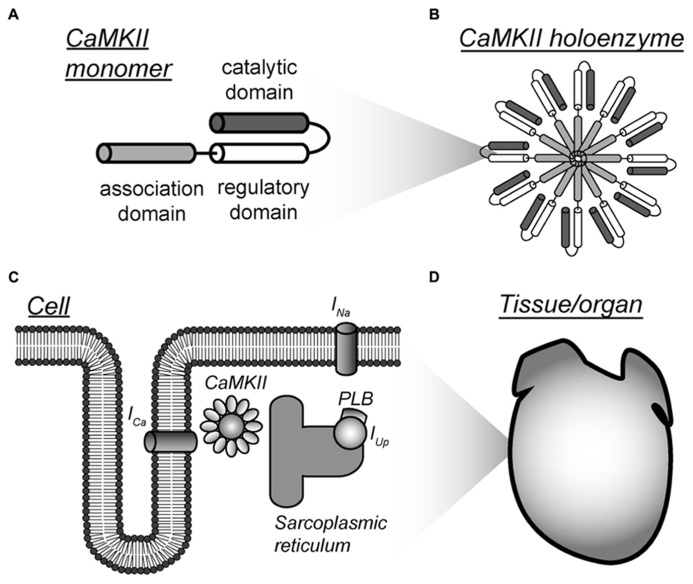
**Multi-scalar mathematical modeling of CaMKII signaling.** **(A–D)** Mathematical modeling has been applied to understand CaMKII function across scales from the molecular to the tissue level. Each scale presents unique challenges and opportunities for modeling efforts. Abbreviations are as follows: *I_Ca_*, L-type Ca^2+^ current; *I_Na_*, fast Na^+^ current; PLB, phospholamban; *I_Up_*, Ca^2+^ uptake into the sarcoplasmic reticulum.

The multifunctional Ca^2+^/calmodulin-dependent protein kinase II (CaMKII) has emerged as an attractive target for systems-based approaches that aim to integrate large experimental data with mathematical modeling and computational approaches across spatial and temporal scales (**Figure 1**). CaMKII serves as a nodal point for a vast signaling network that regulates critical processes like learning and memory, cardiomyocyte contractility, T-cell selection, and expression and localization of class II MHC molecules in dendritic cells (Braun and Schulman, 1995; Maier and Bers, 2002; McGargill et al., 2005; Herrmann et al., 2007; Anderson et al., 2011; Swaminathan et al., 2012). For example, CaMKII regulates multiple important functions in neurons, including synthesis and release of neurotransmitters, modulation of ion channel activity, neurite extension, synaptic plasticity, learning, and gene expression (Braun and Schulman, 1995). Similarly, in heart, CaMKII phosphorylates ion channels, transcription factors, signaling molecules, and other membrane proteins that are critical to cardiac electrical activity and structure. Abnormal CaMKII activity has been observed in human and animal models of cardiovascular disease (e.g., heart failure, myocardial infarction, arrhythmia), and is thought to promote downstream dysfunction in excitation-contraction coupling, structural remodeling, cell death, and even transcriptional activation of inflammation factors (Maier and Bers, 2002; Swaminathan et al., 2012). Current research aims at elucidating how this large effector molecule acts as a pro-cardiac disease/arrhythmogenic molecule and whether it may be effectively targeted for therapy.

Mathematical modeling studies over the past three decades have elucidated important aspects of CaMKII function and signaling mechanisms. Pioneering modeling studies focused on understanding CaMKII structure and function in the brain (Lisman, 1985; Lisman and Goldring, 1988; Hanson et al., 1994; Coomber, 1998; Kubota, 1999; Dupont et al., 2003). This early work motivated later studies that incorporated models of CaMKII activity into models of the whole cell and tissue (mostly cardiac) to understand the larger role of CaMKII signaling in cell/organ function (**Figure 1**; Hund and Rudy, 2004; Iribe et al., 2006; Grandi et al., 2007; Livshitz and Rudy, 2007; Hund et al., 2008; Saucerman and Bers, 2008; Christensen et al., 2009; Koivumaki et al., 2009; Hashambhoy et al., 2010; O’Hara et al., 2011). Recently, these efforts have been expanded to gain insight into the role of CaMKII in human disease (Hund et al., 2008; Christensen et al., 2009; Koivumaki et al., 2009; Hashambhoy et al., 2010; Swaminathan et al., 2011; Lascano et al., 2013; Luo et al., 2013; Zang et al., 2013). This review aims at describing the challenges, advances and opportunities for mathematical modeling of CaMKII signaling at each stage of development across scales from the molecular to the tissue level.

## MODELING THE CAMKII HOLOENZYME

The CaMKII holoenzyme possesses a number of distinguishing characteristics that pose unique challenges for modeling. Briefly (details may be found elsewhere (Couchonnal and Anderson, 2008; Anderson et al., 2011; Swaminathan et al., 2012), multiple CaMKII isoforms are expressed in cells with CaMKIIα and CaMKIIβ expressed predominantly in neurons, whereas CaMKIIγ and CaMKIIδ are more uniformly expressed in other tissues. Structurally, the CaMKII holoenzyme is organized as a hexamer of dimers arranged as two stacked rings. Each monomer is comprised of an N-terminal catalytic domain, a regulatory domain, and a C-terminal association domain. In its inactive conformation, the regulatory domain binds to the active site in catalytic domain, thereby inhibiting the activity of the enzyme. Association of Ca^2+^ bound calmodulin to the regulatory domain causes its release from the active site and exposes the active site in catalytic subunit, enabling the kinase to phosphorylate its substrates (Kolodziej et al., 2000; Rosenberg et al., 2005). Multiple residues within the regulatory domain are also exposed that may subsequently undergo post-translational regulation (e.g., phosphorylation, oxidation, glycosylation) that, in turn, alter kinase function (Lai et al., 1986; Braun and Schulman, 1995; Erickson et al., 2008; Swaminathan et al., 2012; Erickson et al., 2013). Enzyme regulation/activity depends heavily on the multimeric holoenzyme structure (Kolodziej et al., 2000; Hoelz et al., 2003; Rellos et al., 2010; Chao et al., 2011; Stratton et al., 2013). For example, a distinguishing characteristic is the ability of CaMKII to undergo autophosphorylation where an active (Ca^2+^/calmodulin bound) kinase subunit is phosphorylated at a specific residue (Thr286/287) by a neighboring active subunit (Lai et al., 1986; Braun and Schulman, 1995). The autophosphorylated kinase retains activity in the absence of bound Ca^2+^/calmodulin and is thought to contribute to synaptic plasticity and learning functions as well as myocyte excitation-contraction coupling (Silva et al., 1992a,b; Dupont et al., 2003).

One of the most obvious and compelling challenges for modeling of CaMKII is autoregulation. The simplest models consider the entire population of CaMKII subunits that are subject to autophosphorylation at a rate dependent on levels of Ca^2+^/calmodulin (Hanson et al., 1994; Dupont et al., 2003; Gaertner et al., 2004; Chiba et al., 2008). Detailed models have also been developed that incorporate structural information to account for the fact that CaMKII autophophosphorylation is constrained by physical proximity of active subunits (Lisman and Goldring, 1988; Michelson and Schulman, 1994; Zhabotinsky, 2000; Kubota and Bower, 2001; Miller et al., 2005; Lucic et al., 2008; Michalski, 2013). Recently, efforts have been made to also account for other kinase activation modes (e.g., oxidation; Christensen et al., 2009). Modeling studies at the molecular level have generated important insight into CaMKII function. In particular, models have been used to demonstrate that CaMKII activity is sensitive to changes in Ca^2+^ spike frequency and is capable of long-term storage of information at the post-synaptic density by acting as a bistable switch (Lisman, 1985; Lisman and Goldring, 1988; Hanson et al., 1994; Coomber, 1998; Kubota, 1999; Dupont et al., 2003). Furthermore, modeling studies have demonstrated the importance of autophosphorylation for bistability in CaMKII signaling, although there is some debate about the requisite conditions and physiological relevance (Zhabotinsky, 2000; Michalski, 2013). Together, these initial CaMKII modeling studies provided important insight into the link between holoenzyme structure, the ability of the kinase to encode Ca^2+^spike information, and behavior (e.g., long-term potentiation) in neurons. Moreover, this work laid the essential foundation for subsequent multi-scale studies in other systems (e.g., heart).

## MODELING CAMKII SIGNALING IN THE INTACT CELL AND TISSUE

**FIGURE 2:**
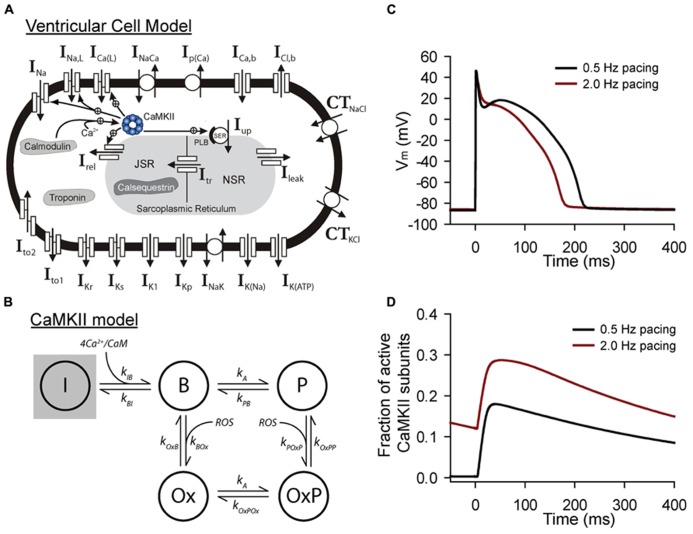
**Mathematical modeling of CaMKII signaling in the intact cell. (A)** Schematic of the Hund-Rudy model of the ventricular action potential that accounts for dynamic CaMKII signaling (Christensen et al., 2009). Abbreviations are as follows: *I_Na_*, fast Na^+^ current; *I_Na,L_*, late (persistent) Na^+^ current; *I_Ca(L)_*, L-type Ca^2+^ current; *I_NaCa_*, Na^+^/Ca^2+^ exchanger; *I_p(Ca)_*, sarcolemmal Ca^2+^ pump; *I_Ca,b_*, background Ca^2+^ current; *I_Cl,b_*, background Cl^-^ current; *CT_NaCl_*, Na^+^/Cl^-^cotransporter; *CT_KCl_*, K^+^/Cl^-^cotransporter; *I_to1_*, transient outward K^+^ current; *I_to2_*, Ca^2+^-dependent transient outward current; *I_Kr_*, rapid delayed rectifier K^+^ current; *I_Ks_*, slow delayed rectifier K^+^ current; *I_K1_*, inward rectifier K^+^ current; *I_Kp_*, plateau K^+^ current; *I_NaK_*, Na^+^/K^+^ ATPase; *I_K(Na)_*, Na^+^-dependent K^+^ current; *I_K(ATP)_*, ATP-sensitive K^+^ current; *I_rel_*, sarcoplasmic reticulum (SR) ryanodine receptor Ca^2+^ release channel; *I_Up_*, SR Ca^2+^ pump; PLB, phospholamban; *I_leak_*, SR Ca^2+^ leak; NSR, network SR; JSR, junctional SR; *I_tr_*, Ca^2+^ transfer from NSR to JSR. **(B)** State diagram for integrated CaMKII model that includes inactive (I), Ca^2+^/calmodulin bound (B), autophosphorylated (P, OxP) and oxidized active states (Ox, OxP). Abbreviations are as follows: *k_IB_, k_BI_*, forward and reverse rate constants, respectively, for transition from inactive state to Ca^2+^/calmodulin bound state; *k_A_, k_PB_*, autophosphorylation and dephosphorylation rate constants, respectively, for transition from Ca^2+^/calmodulin bound state to autophosphorylated state; *k_A_, k_OxPOx_*, autophosphorylation and dephosphorylation rate constants, respectively, for transition from oxidized active state to oxidized and autophosphorylated state; *k_OxB_, k_BOx_*, forward and reverse rate constants, respectively, for transition fromCa^2+^/calmodulin bound state to oxidized active state; *k_POxP_, k_OxPP_*, forward and reverse rate constants, respectively, for transition from autophosphorylated state to oxidized and autophosphorylated active state. Simulated **(C)** action potentials and **(D)** CaMKII activity from the model at two different pacing frequencies to demonstrate sensitivity of CaMKII to pacing frequency.

Much work has been done, particularly in the cardiac field, to incorporate models of the CaMKII signaling pathway into models of the intact cell (**Figure 2**). Modeling of CaMKII signaling at the cellular level poses a unique set of challenges in addition to those encountered at the molecular level (**Table 1**). First, the kinase is sensitive to intracellular Ca^2+^, whose temporal and spatial profile is tightly controlled. In the myocyte, for example, influx of Ca^2+^ through voltage-gated Ca^2+^ channels during the action potential (AP) triggers Ca^2+^ release from the sarcoplasmic reticulum (SR) that leads to a large increase in intracellular Ca^2+^(free and calmodulin-bound) levels. Thus, any cell model of the kinase pathway must address the dynamic nature of the input, namely Ca^2+^-bound calmodulin. Second, once activated, the multifunctional kinase targets a large number of substrates in the cell, from membrane ion channels, pumps and transporters to contractile proteins and even transcription factors. One must consider *a priori* which targets are likely important for the phenomenon of interest. Finally, CaMKII interacts with a vast and complex signaling web that includes other proteins directly regulated by Ca^2+^/calmodulin (e.g., ion channels, calcineurin), protein phosphatases that antagonize CaMKII phosphorylation (e.g., PP1), and other kinases that potentially synergize CaMKII effects (e.g., protein kinase A).

**Table 1:** Challenges for modeling of CaMKII activity across scales from molecule to tissue.

Scale	Challenges for modeling	Representative models
Molecule	Regulation by Ca^2+^/calmodulin and post-translational modification (including autophosphorylation).	Hanson et al. (1994), Coomber (1998), Dupont et al. (2003), Gaertner et al. (2004).
	Complex structure/function relationship.	Lisman and Goldring (1988), Michelson and Schulman (1994), Zhabotinsky (2000), Kubota and Bower (2001), Miller et al. (2005), Lucic et al. (2008), Michalski (2013).
Cell	Dynamic Ca^2+^ signaling as input. Large number of substrates. Resides at center of vast signaling network.	Hund and Rudy (2004), Iribe et al. (2006), Grandi et al. (2007), Livshitz and Rudy (2007), Chiba et al. (2008), Saucerman and Bers (2008), Soltis and Saucerman (2010), Dupont et al. (2010), O’Hara et al. (2011).
	Chronic vs. acute effects of CaMKII activation.	Hund et al. (2008), Koivumaki et al. (2009), Hashambhoy et al. (2010), Lascano et al. (2013), Zang et al. (2013).
Tissue/organ	Chronic and acute remodeling in disease.	Christensen et al., 2009, Swaminathan et al., 2012, Luo et al., 2013.

Despite these numerous obstacles, CaMKII signaling networks have been successfully incorporated with varying degrees of complexity into whole cell models of the myocyte (mostly ventricular) action potential and calcium transient (Hund and Rudy, 2004; Iribe et al., 2006; Grandi et al., 2007; Livshitz and Rudy, 2007; Hund et al., 2008; Saucerman and Bers, 2008; Christensen et al., 2009; Koivumaki et al., 2009; Hashambhoy et al., 2010; O’Hara et al., 2011), as well as other non-cardiac cell types (Dupont et al., 2010; Mironov, 2013). These models have employed different strategies to deal with challenges outlined above. The most common class of models incorporate a scheme where a single population of CaMKII responds to changes in bulk or subspace Ca^2+^/calmodulin (Hund and Rudy, 2004; Iribe et al., 2006; Livshitz and Rudy, 2007; Hund et al., 2008). In other cases, a static formalism is adopted where CaMKII-dependent effects on membrane substrates are implemented in the absence of dynamic changes in CaMKII activity (Grandi et al., 2007; Thiel et al., 2008; Koval et al., 2010; Koval et al., 2012). More recently, consideration has been given to compartmentalization of CaMKII signaling within the cell (Saucerman and Bers, 2008; Song et al., 2008; Soltis and Saucerman, 2010). In general, models account for CaMKII-dependent effects on membrane ion channels and transporters important for Ca^2+^ cycling, including the ryanodine receptor (RyR), SERCA 2a (SR Ca^2+^ ATPase), phospholamban (PLB), and L-type Ca^2+^ channels. As data have emerged regarding CaMKII-dependent effects on other channels important for the action potential (e.g., *I_Na_* and *I_to_*), these effects have also been incorporated (Grandi et al., 2007; Hund et al., 2008; Christensen et al., 2009; Hashambhoy et al., 2010; Koval et al., 2012). It is expected that as we learn more about the specific molecular targets for CaMKII within the cell, models will adapt to account for the new findings.

What have we learned from cellular models of CaMKII signaling? Several computational studies have demonstrated the ability of CaMKII to regulate myocyte action potential, Ca^2+^ transient, and even contractile force in a rate-dependent manner (Hund and Rudy, 2004; Iribe et al., 2006; Livshitz and Rudy, 2007; Soltis and Saucerman, 2010; O’Hara et al., 2011). Interestingly, a role for CaMKII has emerged not only in normal rate dependent behavior (e.g., AP duration adaptation and force-frequency relationships), but also in promoting cellular triggers for arrhythmias such as AP alternans and afterdepolarizations (Grandi et al., 2007; Livshitz and Rudy, 2007; Thiel et al., 2008; Hashambhoy et al., 2010; Koval et al., 2010; Soltis and Saucerman, 2010). Integrated myocyte models have also been applied to increase our understanding of spatial and temporal control of CaMKII signaling (Saucerman and Bers, 2008; Song et al., 2008; Soltis and Saucerman, 2010). Interestingly, studies in this area have demonstrated the importance of affinity for Ca^2+^/calmodulin in defining the differential response of CaMKII and the protein phosphatase calcineurin to the dynamic Ca^2+^ transient (Saucerman and Bers, 2008; Song et al., 2008). Furthermore, studies that incorporate both CaMKII and PKA signaling have shown how the two networks synergize for joint regulation of excitation-contraction coupling (Soltis and Saucerman, 2010). It will be interesting, going forward, to model how other factors such as interaction with scaffolding/anchoring proteins (e.g., β_IV_-spectrin) may contribute to spatial control of CaMKII signaling (Hund et al., 2010; Koval et al., 2010), similar to studies involving other signaling networks (Chan et al., 2012; Greenwald et al., 2014). Finally, although considerable less work has been done in this area compared to smaller scales, progress has been made to understand the role of CaMKII in coordinating function at the tissue/organ level (Livshitz and Rudy, 2007; Christensen et al., 2009; Swaminathan et al., 2011; Luo et al., 2013). These multicellular studies have identified roles for CaMKII in regulating AP heterogeneity and conduction, as well as cardiac pacemaking.

## MODELING CAMKII SIGNALING IN DISEASE

CaMKII plays a critical role in regulating the substrate for both electrical and mechanical dysfunction in cardiovascular disease (Anderson et al., 2011; Swaminathan et al., 2012). Perhaps the greatest challenge for mathematical modeling of CaMKII signaling is how to ultimately link function at the molecular level to behavior at cell/tissue level in the setting of disease. Among the difficulties for modeling in this area involves distinguishing between acute and chronic effects of CaMKII activity. For example, while acute effects of CaMKII are mostly mediated by posttranslational modification of substrates, chronic CaMKII activation may facilitate large scale remodeling changes due to effects on transcription and gene expression (Koivumaki et al., 2009; Swaminathan et al., 2012). Mathematical modeling and computer simulation have been used to generate new insights into molecular mechanisms for arrhythmia in several disease states, including myocardial ischemia/infarction, heart failure, and diabetes (Hund et al., 2008; Christensen et al., 2009; Swaminathan et al., 2011; Lascano et al., 2013; Luo et al., 2013; Zang et al., 2013).

Arrhythmia mechanisms in the canine infarct border zone have been studied extensively using a mathematical modeling approach (Cabo and Boyden, 2003; Cabo et al., 2006; Hund et al., 2008; Christensen et al., 2009). The canine infarct border zone is particularly well suited to mathematical modeling approach due to the tremendous amount of available data at the molecular, cellular, and tissue level (Pinto and Boyden, 1999). Mathematical models have been used to link defects in CaMKII signaling with ion channel remodeling, abnormal Ca^2+^ handling, and arrhythmias in the infarct border zone. Specifically these studies have demonstrated that increased autophosphorylation and oxidation of the kinase results in increased activity that both increases Ca^2+^ leak from the sarcoplasmic reticulum and compromises availability of voltagegated Na^+^ channels to create a favorable substrate for arrhythmias (Hund et al., 2008; Christensen et al., 2009). More recently, mathematical models have been used to study the role of chronic CaMKII activation in sinus node dysfunction in the setting of heart failure and diabetes (Swaminathan et al., 2011; Luo et al., 2013). A two dimensional model of the intact sinus node has been applied to demonstrate that CaMKII-induced apoptosis and associated loss of sinoatrial node cells disrupts the source–sink balance between the sinoatrial node and surrounding atrial myocardium resulting in slowed pacemaking and even failure (Swaminathan et al., 2011; Luo et al., 2013). Other studies have used mathematical modeling to determine relative importance of direct CaMKII effects and compensatory changes in gene regulation in the setting of chronic CaMKII overexpression (Koivumaki et al., 2009). Finally, in addition to common forms of acquired disease (e.g., myocardial infarction, heart failure, diabetes), mathematical models have been used to better understand the role of CaMKII in congenital disease (Thiel et al., 2008; Koval et al., 2012). A recent study used mathematical modeling to demonstrate that human variants identified in the CaMKII phosphorylation motif of Na_v_1.5 confer arrhythmia susceptibility by mimicking the phosphorylated channel (Koval et al., 2012), while an earlier study examined the role of CaMKII regulation of SR Ca^2+^ release in increased incidence of afterdepolarizations in Timothy syndrome (Thiel et al., 2008). Together these studies demonstrate the potential for mathematical modeling and computer simulation in advancing our understanding of CaMKII biology and its role over a broad range of cardiovascular disease.

## FUTURE DIRECTIONS

This review has outlined the many unique challenges and opportunities for multiscale mathematical modeling of CaMKII signaling. While great strides have been made in development and application of mathematical models of CaMKII signaling from molecule to tissue, clearly there are outstanding issues and unanswered questions to be addressed by future research in this area. At the molecular level, the recent discovery of the CaMKII crystal structure represents an exciting development with great potential for modeling (Chao et al., 2011). Similarly, it will be important for future modeling efforts to address novel pathways for regulation of CaMKII activity (e.g., glycosylation). At the cell level, a daunting challenge remains the sheer number of targets for CaMKII within the cell, with new substrates identified every year. Moreover, it remains to be understood the “tipping point” from the adaptive to the maladaptive aspects of CaMKII signaling. Finally, while most models have focused on the ventricular myocyte as a system, clearly CaMKII has important roles in other heart regions/cell types (e.g., atrial, sinoatrial node cells). Models of these different cell types that incorporate cell-specific CaMKII signaling will be of great use for studying CaMKII signaling at the organ level.

## Conflict of Interest Statement

The authors declare that the research was conducted in the absence of any commercial or financial relationships that could be construed as a potential conflict of interest.
